# Reduced Sensitivity to Immediate Reward during Decision-Making in Older than Younger Adults

**DOI:** 10.1371/journal.pone.0036953

**Published:** 2012-05-24

**Authors:** Ben Eppinger, Leigh E. Nystrom, Jonathan D. Cohen

**Affiliations:** 1 Princeton Neuroscience Institute, Princeton University, Princeton, New Jersey, United States of America; 2 Center for the Demography of Aging, Woodrow Wilson School of Public and International Affairs, Princeton University, Princeton, New Jersey, United States of America; 3 Max-Planck Institute for Human Development, Berlin, Germany; University College London, United Kingdom

## Abstract

We examined whether older adults differ from younger adults in the degree to which they favor immediate over delayed rewards during decision-making. To examine the neural correlates of age-related differences in delay discounting we acquired functional MR images while participants made decisions between smaller but sooner and larger but later monetary rewards. The behavioral results show age-related reductions in delay discounting. Less impulsive decision-making in older adults was associated with lower ventral striatal activations to immediate reward. Furthermore, older adults showed an overall higher percentage of delayed choices and reduced activity in the dorsal striatum than younger adults. This points to a reduced reward sensitivity of the dorsal striatum in older adults. Taken together, our findings indicate that less impulsive decision-making in older adults is due to a reduced sensitivity of striatal areas to reward. These age-related changes in reward sensitivity may result from transformations in dopaminergic neuromodulation with age.

## Introduction

Older adults face important decisions regarding matters such as how to spend their pension savings or decisions regarding healthcare and nursing home placement. These decisions often involve trade-offs between short term and long-term benefits, e.g., the short-term benefit of staying at home compared to the potential long-term benefit of having convenient access to health care and nursing services in a retirement home. Given the rising proportion of elderly in most western societies, the outcome and quality of these decisions have an increasing economic and political impact [1], [2]. However, despite the importance of this topic, there is a surprising lack of empirical research on age-related differences in decision-making. Moreover, the neurophysiological mechanisms that may underlie age-related changes in choice behavior are largely unexplored [3], [4].

In this study we were interested in whether older adults differ from younger adults in the degree to which they favor immediate over delayed rewards during decision-making. To examine the neural correlates of age-related differences in delay discounting, we employed functional magnetic resonance imaging (fMRI) while younger and older adults made decisions between smaller but sooner and larger but later monetary rewards.

Delay discounting refers to the devaluation of rewards as a function of the time of their delivery [5]. For example, many individuals prefer receiving $20 immediately to receiving $30 in two weeks. Several theoretical accounts have been proposed to explain delay discounting [6], [7], [8]. One influential approach, the so-called two-systems model, suggests that two interacting systems are involved when participants make intertemporal decisions: 1) an impulsive (i.e., high discounting) system – the so-called beta (β-) system – that is primarily activated by choice options involving immediately available rewards; and 2) a more patient system – the delta (δ-) system – that is generally active during decision-making and more sensitive to longer term rewards [7].

Neurophysiological evidence for the two-systems model comes from functional imaging (fMRI) studies on delay discounting. McClure and colleagues (2004) showed that neural activity consistent with the function of the β-system (i.e., responsiveness primarily to immediate rewards) is observed in limbic structures, such as the striatum, and their projection areas in the ventromedial prefrontal and posterior cingulate cortex. In contrast, neural activity consistent with the δ-system is observed in areas that are commonly associated with cognitive control, such as the lateral prefrontal and posterior parietal cortex [9]. However, it should be noted that recent studies questioned this view by showing that the limbic system is not exclusively sensitive to immediate reward [8], [10]. These findings suggest that a component of activity in limbic areas may reflect the subjective value of reward independently of delay [8], [10], [11], [12].

Interestingly, delay discounting is characterized by large individual differences (for a review see [13]). Increased delay discounting is observed during adolescence, which is consistent with findings pointing to enhanced limbic responsiveness during this developmental period (e.g. [14], [15]). Similar to the findings in adolescents, functional MRI in healthy younger adults has revealed a positive correlation of delay discounting with fMRI activity in the ventral striatum [16], [17]. In contrast, addiction is typically associated with increased discounting but reduced limbic responses to reward (see [13]). This may be due to the effects of chronic abuse of drugs affecting the mesolimbic dopaminergic system.

Together, these results suggest that a preference for immediate reward – that is, more impulsive decision-making – is related to an increased sensitivity of limbic areas to reward [9], [17]. These areas are highly innervated by dopaminergic projections from the midbrain, suggesting that individual differences in impulsivity might depend on the degree of dopaminergic neuromodulation [18].

Only a few behavioral studies so far have investigated age-related differences in delay discounting in older adults. Results of these studies have shown that older adults discount rewards less than younger adults [19], [20], [21]. However, evidence for age-related reductions in discounting is not unequivocal. In a follow-up study of their original results Green and colleagues (1996) showed that discount rates also co-vary with income level [22]. That is, lower income older adults discount rewards more steeply than upper income older and younger adults. Current findings suggest that discount rates also correlate positively with fluid intelligence and that this effect may be partially mediated by activity in the anterior prefrontal cortex [23]. To date it is unclear whether and how the relation between fluid intelligence, income and discounting change as function of age.

The neurophysiological mechanisms underlying age-related differences in discounting also remain unclear. However, there is increasing evidence that age-related transformations in neuromodulatory systems, especially in the midbrain dopamine system, may play an important role [3], [4]. Most of the evidence for this view comes from fMRI studies on age-related differences in reward processing and reward-based learning. Results of these studies suggest that age-related impairments in learning are associated with reduced striatal activity during the anticipation and processing of rewards in older adults [24], [25]. These results are consistent with findings from a recent multimodal imaging study, which showed that age-related differences in fMRI activations during reward processing might result from reduced dopamine receptor stimulation as measured with positron emission tomography (PET) [26]. Taken together, the current literature points to the important role of age-related changes in dopaminergic neuromodulation for reward processing and decision-making in older age.

The aim of this study was to test the hypothesis that older adults exhibit reduced delay discounting, and that this is related to diminished activity in limbic structures that are major targets of dopaminergic neuromodulation and are selectively responsive to immediate rewards (i.e., thought to mediate steep discounting). To do so, we acquired functional MR images while participants performed a delay-discounting task. We expected reduced delay discounting for older than younger adults [19], [20], [21]. Consistent with previous results we predicted that choice pairs involving immediate rewards would activate (para-) limbic areas such as the striatum, ventromedial prefrontal and posterior cingulate cortex. In contrast, areas associated with cognitive control and shallower discounting, such as the lateral prefrontal cortex and posterior parietal cortex, should be consistently engaged in all decision-making trials [9]. Finally, we predicted that reduced delay discounting in older adults should be associated with decreased activity in limbic areas, which are most sensitive to immediate reward.

## Methods

### Participants

Seventeen younger adults (undergraduate and graduate students from Princeton University) and 15 older adults (recruited from the local Princeton, New Jersey community) participated in the study. Two younger adults were excluded because of excessive head motion. The effective sample consisted of 15 younger adults (mean age = 20.9 SD = 3.0, 9 male, age range 18–28) and 15 older adults (mean age = 69.7, SD = 4.4, 8 male, age range 65–80), all right-handed. Participants gave written informed consent. The Institutional Review Board of Princeton University approved the study. The participants completed a biographical questionnaire and several psychometric tests (Digit–Symbol Substitution test, DSS; Raven’s Progressive matrices; Spot-the-Word test [27], [28], [29]. The psychometric results revealed age-related reductions in fluid intelligence as reflected in lower DSS and Raven’s scores for older than younger adults (both p<.002, η^2^ >.29). In contrast, higher scores on the Spot-the-word test (p<.01, η^2^ = 20) for older than younger adults indicate age-related improvements in crystallized intelligence [30].

### Task

Participants made two-choice decisions between an earlier and smaller monetary reward and a later and larger monetary reward. The earlier reward option was always presented on the left side of the screen and varied randomly between $7.50 and $35.00. The percent difference in dollar amounts between the two rewards was drawn randomly from six values (1%, 5%, 15%, 25%, 35%, 50%). The delay to the early reward was set to be either today, in two weeks, or in four weeks. The delay between the early reward and the late reward was either two weeks or four weeks. Cases in which the late reward would have been received more than six weeks from the date of the experiment were eliminated.

### Procedure

The participants performed a behavioral and an fMRI session. In the behavioral session participants were screened for MR eligibility and performed the psychometric tests. If they were eligible for MR imaging they were invited back to perform the monetary decision-making task in the MR scanner. On each trial, participants were asked to make a decision between a smaller but sooner reward and larger but later reward. They were instructed that there were no correct answers and that at the end of the experiment they would receive one of their choices as compensation. This choice was randomly selected from their choice set and the respective amount of money was paid to them in form of a check, which was delivered at the time point corresponding to their choice.

During fMRI scanning participants performed a total number of 120 choices. The task was paused every 30 trials to allow participants to rest. Each choice pair was presented for 8 seconds. If the choice was made within 6 seconds after stimulus onset a green triangle occurred underneath the preferred choice to indicate that the choice was registered. If no response was given within 6 seconds both triangles turned red for 2 seconds to indicate a timeout. Following the stimulus a blank screen was displayed for a variable interval of 2–8 seconds. The inter-trial interval (ITI) varied between 10 and 16 seconds, according to a long-tailed exponential distribution (λ = 4.0, mean ITI = 12.7 seconds) [31].

The stimuli were projected on a screen mounted at the rear of the scanner bore, which participants viewed through a series of mirrors. Stimuli were presented using the software E-Prime (PST Inc., Pittsburgh, PA) and manual responses were registered using an MR-compatible button box. A pillow and foam cushions were placed inside the head coil to minimize head movements.

### fMRI Data Acquisition

MRI data acquisition was performed using a head-dedicated 3 Tesla MRI scanner (Allegra; Siemens, Erlangen, Germany) at Princeton University. At the beginning of the fMRI session high-resolution (1 mm^3^) T1-weighted structural images were acquired using an MP-RAGE pulse sequence [160 axial slices; field of view (FOV), 256; repetition time (TR), 2500 ms; echo time (TE), 4.38 ms; flip angle, 8°]. AC-PC aligned functional images were acquired using a T2* weighted EPI sequence [33 interleaved slices; FOV, 192; TR, 2000 ms; TE, 30 ms; flip angle, 90°].

### Behavioral Data Analysis

Choice behavior was analyzed using Matlab (MATLAB, Mathworks Inc, Natick, MA) and SAS (SAS Institute Inc, Cary, NC). The dependent measures were percentage of delayed choices and reaction time (RT). Age-related differences in delay discounting were analyzed using repeated measures ANOVAs with the factors of age group (younger, older adults), delay (today –2 weeks, today –4 weeks, 2 weeks –4 weeks, 2 weeks –6 weeks and 4 weeks –6 weeks), and percent reward difference (1%, 5%, 15%, 25%, 35%, 50%). For correlation analyses we computed a measure of discounting by subtracting the percentage of delayed choices on choice pairs that involve a 4-week delay (today-4weeks, 2weeks-6weeks) from the percentage of delayed choices on choice pairs that involve a 2-week delay (today-2weeks, 2weeks-4weeks).

### FMRI Data Analysis

FMRI data analyses were performed using AFNI [32] and SPM (SPM8; Welcome Department of Imaging Neuroscience, London, UK).

#### Preprocessing

Functional data were slice-time corrected to the second volume using Fourier interpolation and realigned using rigid-body 3D motion correction (AFNI). Transient spikes in the EPI data were removed with the AFNI program 3dDespike. Percent signal change was calculated for each voxel with respect to the mean activation across the time series.

#### Spatial normalization

To avoid a normalization bias towards the anatomy of younger adults [33], [34] the fMRI images were registered to a study-specific grey matter template using DARTEL toolbox in SPM8 [35]. We first registered the functional images to the high-resolution T1 image using a local Pearson correlation cost function [36]. Functional and structural images of each participant were manually aligned with the SPM tissue probability maps to provide an approximate alignment across participants. The structural images were segmented into their tissue components using the unified segmentation procedure. The resulting grey and white matter images were used during the DARTEL procedure to create the study-specific template [35], [37]. The procedure results in flow-fields that parameterize the non-linear deformations that are applied to match each individual image to the template. These flow-fields were used to normalize the smoothed (Gaussian FWHM 8 mm) EPI data to an MNI-registered version of the template image.

#### Deconvolution analysis

To control for age-related differences in the shape of the hemodynamic response function (HRF) we used the deconvolution approach as implemented in AFNI. We estimated the HRF within each individual, condition and voxel over a period of 16 seconds after stimulus onset using eight piecewise linear B-splines or “tent” functions [38]. Statistical analyses were performed on the mean beta-coefficients of the HRF (averaged from 4–8 seconds after stimulus onset). Similar to previous studies [9], [39] two sets of regressors were used for the analyses. The first regressor (the β-regressor) weighs choice pairs involving immediate reward with 1 and all other choice pairs as well as baseline periods with 0. The second regressor (the δ-regressor) weighs all choice pairs (involving immediate and delayed earlier reward) with 1 and baseline periods with 0. Reaction time was included into the model as a parametric regressor (see Figure S1). Baseline drifts were modeled using a third degree polynomial function and motion correction parameters were included as nuisance variables. The beta-coefficients from the deconvolution analyses were analyzed using a whole-brain mixed effects ANOVA with the factors of age group (younger vs. older adults), choice option (immediate vs. all choice options) and the random factor, subjects. The contrasts of interest in this ANOVA were the regression weights for the β-regressor versus those for the δ-regressor, and the weights for the delta regressor vs. 0. This ANOVA goes one step beyond the multiple regression analysis used in previous studies [9], [39]. We used this strategy to identify the regions showing the strongest selective responses to choices involving immediate rewards, in order to maximize sensitivity to age-related differences in such regions. For comparison, we also report results of the regression analysis used in the McClure et al. studies [9], [39]. As shown in Figure S2a similar, although less pronounced effects in the beta system were obtained using this analysis. The difference in the relative involvement of the two systems between studies may be due to shallower discounting and/or reduced overall power in the imaging data in the current study. Importantly, age-related differences in ventral striatal activations for immediate reward are observed independently of the applied contrast (see Figure S2b). To correct for multiple comparisons we used the AFNI program AlphaSim and determined that a corrected (family-wise) p-value of .05 is achieved with a minimum cluster-size of 48 voxels, each significant at p<.001. To further investigate age differences in immediacy effects in the ventral striatum we performed a follow-up analysis using an anatomical ROI corresponding to the nucleus accumbens as defined using Talairach atlas (as implemented in AFNI). Percent signal change in nucleus accumbens was analyzed using an ANOVA involving the factors age group and choice option.

## Results

### Behavioral Data

#### Choice behavior

**Figure 1:**
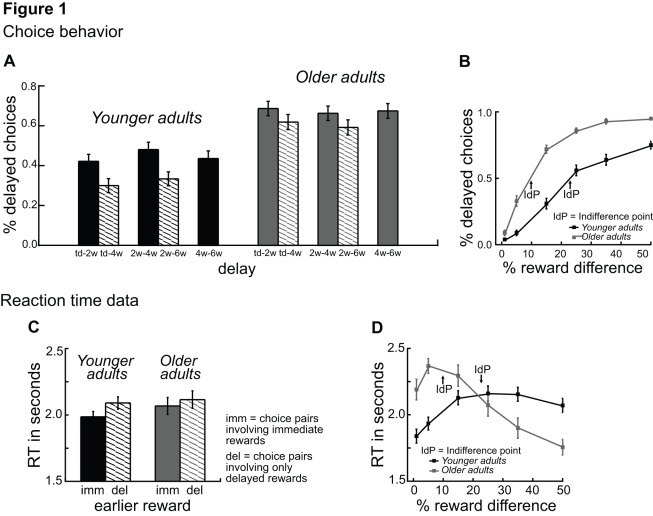
Age differences in choice behavior and reaction times. A) Percentage of delayed choices (y-axis) as a function of delay (x-axis), displayed separately for younger (black) and older adults (grey). B) Percentage of delayed choices (y-axis) as a function of the % difference between rewards, displayed separately for younger (black) and older adults (grey). C) Reaction time in seconds (y-axis) for choice pairs involving immediate rewards (solid) and choice pairs involving only delayed rewards (dashed) (x-axis), displayed separately for younger (black) and older adults (grey). D) Reaction time in seconds (y-axis) as a function of the % difference between rewards, displayed separately for younger (black) and older adults (grey).

The analysis of the choice behavior (% delayed choices) revealed significant main effects of age group F(1, 28) = 11.51, p<.002, η^2^ = .29, and delay F(4, 112) = 14.67, p<.001, η^2^ = .34, ε = .84, as well as a significant age group x delay interaction F(4, 112) = 3.07, p<.02, η^2^ = 07, ε = .81. As shown in Figure 1a, older adults chose delayed rewards more frequently than younger adults. Furthermore, older adults showed reduced effects of delay, suggesting that they discount rewards less than younger adults. The analysis also revealed a significant main effect of percent reward difference, F(5, 140) = 107.54, p<.001, η^2^ = .77, ε = .45 and an interaction between age group and reward difference, F(5, 140) = 3.43, p<.03, η^2^ = .02, ε = .45. Separate analyses for each of the percent difference values showed a significantly larger percentage of delayed choices for older than younger adults for percent differences greater than 15%, (all p<.008 η^2^ >.29, see Figure 1b). These results show that older adults switch earlier from choosing the immediate reward to choosing the delayed reward (they have lower indifference points) than younger adults. These findings are supported by an analysis of the indifference points (IDPs). In this analysis we fitted a logistic regression to each individual’s choice data (separately for each of the delays) and extracted the point at which participants were indifferent between choosing immediate and choosing delayed rewards. An ANOVA on the IDPs revealed lower indifference points for older adults (M = 13.3, SE = 1.4) than younger adults (M  = 26.8, SE  = 2.1) (p<.01, η^2^  = .28, see Figure 1b).

To examine whether individual differences in preferences might affect age differences in choice behavior and the associated fMRI results we performed a subgroup analysis (see Figure S4a). In this analysis we matched nine participants of each age group for discounting behavior (the difference in % delayed choices between four week and two week delays). An ANOVA with the factors age group and delay showed a significant main effect of age group (p<.02, η^2^ = .31), as well as a main effect of delay (p<.001, η^2^ = .67), but no significant interaction between age group and delay (p  = .52). Thus, the matched subgroups differed in their overall tendency to choose for delayed reward, but they did not differ with respect to discounting of delayed reward (see Figure S4a).

**Reaction time data.** The analysis of the reaction time data revealed a significant main effect of delay F(4, 112)  = 3.24, p<.01, η^2^  = .10, ε  = .90, which reflects the longer RT for choice pairs involving only delayed rewards than for choice pairs involving immediate rewards (see Figure 1c). Moreover, we found a significant interaction between age group and percent reward difference F(5, 140)  = 4.51, p<.01, η^2^  = .13, ε  = .40. As shown in Figure 1d, reaction time decreased as a function of the distance from indifference point in both younger and older adults, suggesting that decision difficulty was greater when the options were closest to participants’ indifference points.

**Figure 2:**
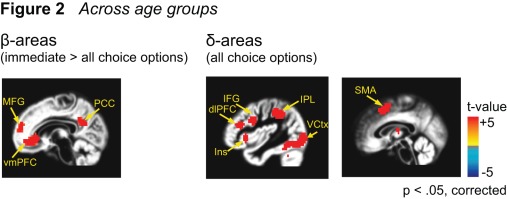
Activity in β-areas and δ- areas across age groups. Left: Significant activations (t-statistics) for choice pairs involving immediate options (β-areas) averaged across age groups. Talairach coordinates: MFG: -8, 57, 19; vmPFC: 0, 39, -4; PCC: 2, −52, 31. Right: Significant activations for all choice pairs (δ- areas). Activations are significant at p<.05, corrected for multiple comparisons. Talairach coordinates: dlPFC: −42, 36, 22; IFG: −45, 7, 32; Ins: −35, 22, 3; SMA; −1, 15, 48; IPL: 30, −48, 40.

**fMRI data.** The fMRI analysis showed areas of cortical activity that was significantly greater for choice pairs involving immediate reward in regions previously identified with the β-system: medial prefrontal cortex (MFG), ventro-medial prefrontal cortex (vmPFC) and posterior cingulate cortex (PCC) (all t >3.5, p<.05) (see Figure 2). We also obtained activity in the ventral striatum however this effect was modulated by age (see below). These findings replicate those of previous studies showing activations for immediate reward in the medial prefrontal and ventro-medial prefrontal cortex (see also Figure S2a). Similarly, we replicated the pattern of cortical activity for areas previously identified with the δ-system: Areas showing a response for all choice options included dorsolateral prefrontal cortex (dlPFC) and inferior parietal lobe (IPL) (see Figure 2; for the corresponding time courses please refer to Figure S3).

**Figure 3:**
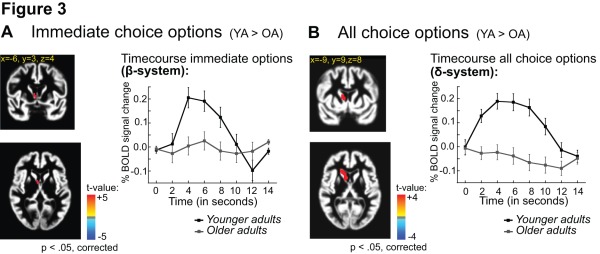
Age differences in striatal activity. A) Left: Significant main effect of age group for choice options involving immediate reward in the ventromedial caudate (t-statistics, significant at p<.05, corrected for multiple comparisons). Right: Time course of BOLD signal change (on the y-axis) for younger (black) and older adults (grey). The x-axis shows time post stimulus onset in seconds. The coordinates refer to Talairach space. B) Left: Significant main effect of age group in the dorsal striatum for all choice options (t-statistics, significant at p<.05, corrected for multiple comparisons). Right: Time course of BOLD signal change (on the y-axis) for younger (black) and older adults (grey). The x-axis shows the repetition time (TR) in seconds. The x-axis shows time post stimulus onset in seconds. The coordinates refer to Talairach space.

Task-related activity was also observed in subcortical areas. The analysis revealed a significant main effect of age group in the dorsal striatum (t  = 3.7, p<.05). As shown in Figure 3b, older adults showed reduced activations in the dorsal striatum than younger adults.

**Figure 4:**
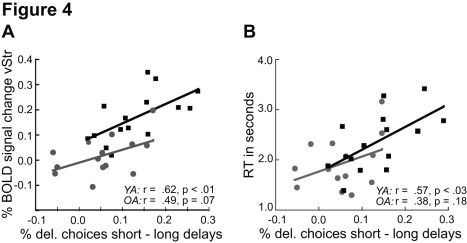
Correlation analyses. A) Correlation between delay discounting (% delayed choices short – long delays) (x-axis) and % BOLD signal change for immediate choice options in the ventral striatum (y-axis). Younger adults are shown in black, older adults are shown in grey. B) Correlation between delay discounting (x-axis) and reaction time for choice options involving delayed reward (y-axis). Younger adults are shown in black, older adults are shown in grey.

Most interestingly, we found a significant interaction between age group and choice options (immediate vs. all choice options) in the ventral striatum, F(1, 28)  = 13.17, p<.05. Follow-up analyses showed significantly reduced activity in ventral striatum for immediate choice options in older than younger adults (t  = 3.6, p<.05, see Figure 3a). As shown in Figure S2b, qualitatively similar results were obtained for the contrast between immediate and all other choice options (t  = 3.6, p<.05). To further examine the relationship between age-related differences in ventral striatal activations and age-related changes in choice behavior and RT, we performed a correlation analysis. We obtained significant positive correlations between ventral striatal activity and delay discounting for younger (r  = .62, p<.01), but not for older adults (r  = .49, p  = .07) (see Figure 4a). Moreover, the analysis revealed a significant positive correlation between discounting and reaction time on delayed but not immediate choices, once again for younger but not older adults (see Figure 4b). Please note that we had to exclude one older adult as an outlier (>3 SD from the mean). Inclusion of the outlier leads to even more pronounced age-related differences in the correlations between discounting and striatal activity (older adults: r = −.19, p = .49).

**Figure 5:**
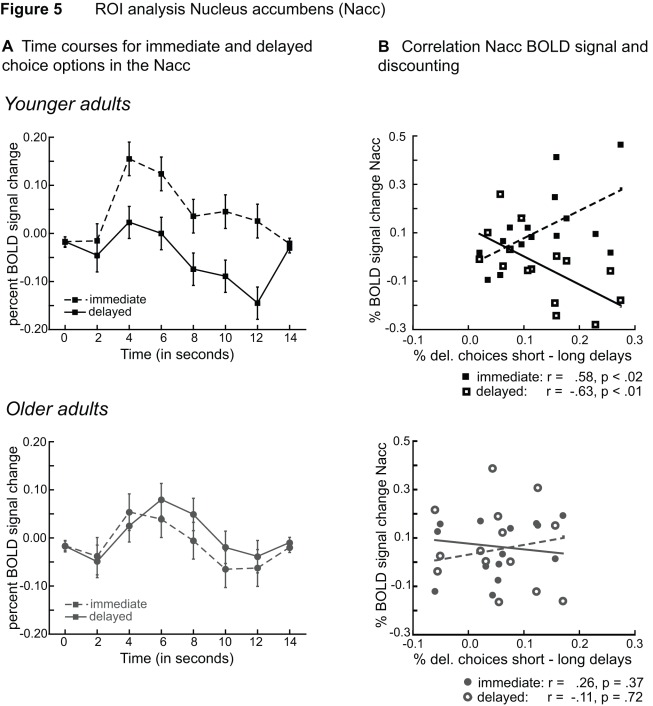
Regions of interest (ROI) analysis of BOLD activity in the nucleus accumbens. A) Time courses for immediate (dashed) and delayed (solid) choice options in the nucleus accumbens (Nacc, as defined using Talairach atlas). Younger adults (top) are shown in black, older adults (bottom) are shown in grey. B) For younger adults (top) BOLD signal change for immediate choice options in the nucleus accumbens correlates positively with discounting. In contrast, BOLD signal change for delayed choice options correlates negatively with discounting. For older adults (bottom) no significant correlations are obtained.

As shown in Figure 3a the observed age effect in BOLD activity for immediate choice options is not exactly located in the ventral striatum but rather reflects activity close to the most ventro-medial part of the caudate. To further examine age differences in ventral striatal immediacy effects we performed an ROI analysis of activity in the nucleus accumbens. This analysis showed a significant interaction between age group and choice option, (p<.03, η^2^ = .16). Separate analyses for the two age groups showed a significant difference in signal change between immediate and delayed options in the ventral striatum for younger adults (p<.03, η^2^ = .29), but not for older adults (p  = .43) (see Figure 5a). In analogy to the whole brain analysis we also examined correlations between BOLD activity for immediate options and choice behavior. For younger adults BOLD signal change for immediate choice options in the nucleus accumbens correlates positively with discounting (r  = .58, p<.02). In contrast, BOLD signal change for delayed choice options correlates negatively with discounting (r = −.63, p<.01). For older adults no significant correlations are obtained (p’s >.37), (see Figure 5b).

## Discussion

Both younger and older adults constantly face trade-offs between choice options involving immediate benefits and ones involving (often larger) future benefits. Despite having an expectation of fewer remaining years of life, older adults have been suggested to be more patient in these types of decisions than younger adults [19], [20], [21]. To examine the neural mechanisms that underlie these changes in decision-making, we used a delay-discounting task in younger and older adults and acquired functional MR images. More specifically, we sought to confirm the observation that older adults favour immediate rewards less than younger adults, and to test the hypothesis that this is related to a diminished response of the proposed beta system to immediate rewards among older adults.

Consistent with our predictions, the behavioral results showed an increased percentage of delayed choices in older than younger adults. Moreover, older adults switched earlier from choosing the immediate reward to choosing the delayed reward (they show lower indifference points) than younger adults. This indicates that they are willing to wait for smaller differences between rewards (see Figure 1b). Most interestingly, we found reduced delay effects in older adults, indicating that they discount rewards less than younger adults (see Figure 1a). These findings are consistent with the results of previous behavioral studies and point to a reduced immediacy bias and a stronger willingness to wait for rewards among older adults [19], [20], [21].

It is worth noting that recent findings also point to positive associations between income level and discounting, as well as fluid intelligence and discounting. Hence it cannot be ruled out that factors other than age contribute to reduced discounting in the older age group. Income levels are generally difficult to compare between college students and the elderly. However, the fact the younger adults were students at Princeton University and the older adults were recruited from the local New Jersey community renders it less likely that differences in socio-economic status contributed to the current behavioral findings. The higher Raven scores in younger than older adults suggest that differences in fluid intelligence did not account for the results we obtained. Thus, it remains likely that the reduction in discounting we observed for older adults reflects a reduced immediacy bias, which may be due to changes in sensitivity to immediate reward.

An analysis of the reaction time data revealed increased latencies for choice options involving only delayed rewards compared to choice options involving immediate rewards (see Figure 1c). This suggests that choice options involving only delayed rewards are associated with increased decision difficulty and hence higher levels of cognitive control. Moreover, we found that RT increased as a function of the proximity of the choice pairs to the indifference point in younger and older adults (see Figure 1d). This result is consistent with findings that point to increased decision conflict when participants are indifferent with respect to their choice behavior.

In the fMRI analyses we focused on the comparison of two neural systems previously found to be involved in delay discounting: an impulsive (β)-system that is preferentially activated by choice options involving immediate rewards, and a more patient (δ)-system that is engaged more generally by cognitive control and decision-making [7], [9]. Consistent with previous studies, we found significant activations for choice pairs involving immediate rewards in areas previously associated with the (β)-system, such as medial prefrontal cortex (MFG), ventro-medial prefrontal cortex (vmPFC) and posterior cingulate cortex (PCC) (see Figure 2). Moreover, we found that areas implicated in cognitive control processes and previously associated with the (δ)-system were active during all decision-making trials [9], [40], including dorsolateral prefrontal cortex (dlPFC), inferior frontal cortex (IFG), and inferior parietal lobe (IPL) (see Figure 2). Taken together, our data replicate previous findings suggesting that two separable neural systems may be involved in intertemporal choice [9].

Consistent with our predictions, we found no age-related differences in BOLD activity in the major components of the (δ)-system such as the dlPFC, IPL, Insula and SMA as well as cortical areas associated with the (β)-system such as the ventromedial PFC and posterior cingulate (see Figure S3).

In contrast, we obtained significant age-related differences in BOLD activity in subcortical areas associated with the (β)-system. Older adults showed reduced activity in the dorsal striatum for all choice options compared to immediate choice options (see Figure 3b). This is consistent with their greater overall tendency to choose delayed rather than immediate rewards (see Figure 1a). The dorsal striatum plays an important role in reward prediction and reinforcement learning and is highly innervated by dopaminergic projections from the midbrain [41]. Consistent with these findings, the present results suggest a reduced dorsal striatal sensitivity to reward in older adults. More research is needed to clarify the role of the dorsal striatum in decision-making and to identify experimental factors that modulate age-related differences in the dorsal striatum during delay discounting.

Most interestingly, we also observed significant age-related differences in activations in subcortical (β)-related areas associated with immediate reward options. Older adults showed reduced activity in the ventral striatum for immediate reward options compared with younger adults (see Figure 3a). It should be noted that the age effects shown in Figure 3a are not exactly within the ventral striatum but rather reflect activity close to the most ventro-medial part of the caudate. This may be due to age-related reductions of grey matter in this region, which lead to a bias in the localization of age-related differences in activations. A region-of-interest (ROI) analysis in the nucleus accumbens (see Figure 5a) showed similar (although less pronounced) reductions of BOLD activity for immediate choice options in older than younger adults. Moreover, in support of our claim of a functional association of activity in ventral striatum and discounting, we found a significant correlation between activity in this area and delay discounting in younger adults (see Figure 4a). That is, stronger activity in the ventral striatum was associated with the more impulsive choice behavior that we observed in the younger adults [17]. No significant correlation between discounting and ventral striatal activity was obtained in the older age group, indicating that activity in this area is less closely associated with discounting behavior in older adults. It should be noted that we did not obtain significant correlations between age and discounting (YA: r = −18, p = .53; OA: r = .03, p  = .93) or between age and ventral striatal activity (YA: r  = −.11, p  = .71; OA: r  = .23, p  = .41) *within* the two age groups. Hence, it is unlikely that the present findings are due to an overactive ventral striatal system in a subset of younger participants as has been observed using similar tasks in adolescents (e.g. [42]).

The results of the ROI analysis in the nucleus accumbens further support these conclusions. For younger adults we obtained a significant positive correlation between discounting and ventral striatal BOLD activity for options involving immediate reward. For delayed reward options the reverse pattern was observed (a significant negative correlation; see Figure 5b). This finding may indicate that individuals with a greater bias to immediate reward devalue options that only involve delayed reward. In line with the results of the whole brain analysis, for older adults we did not obtain significant correlations between discounting behavior and activity in the nucleus accumbens (see Figure 5).

Taken together, these findings are consistent with the hypothesis that less impulsive decision-making in older adults is associated with reduced activity in the ventral striatum to immediate reward. Recent findings from a PET study suggest a potential mechanism by which changes in dopaminergic neuromodulation in the ventral striatum can affect individual differences in impulsivity [18]. Results from this study indicate that higher trait-levels of impulsivity are associated with reduced D2 autoreceptor availability in the midbrain as well as increased dopamine release in the ventral striatum. Given these findings, the present results suggest that an age-related decrease in impulsivity during decision-making might result from reduced dopaminergic neuromodulation in the ventral striatum. Such an interpretation is consistent with results of several developmental studies that point to steeper discount rates and a progressive maturation of the limbic and prefrontal circuits involved in delay discounting from childhood through adolescence to early adulthood [43], [44].

It should be noted however that the broader literature on age-related differences in the neural correlates of reward processing includes some inconsistencies. For example, findings by Samanez-Larkin and colleagues point to a preservation of striatal activity during the anticipation of reward in older adults in the monetary incentive delay (MID) task [45]. More recent findings from these authors suggest that suboptimal decision-making in a financial investment task may be partly mediated by increased temporal variability of activity in the ventral striatum [46]. How increased variability in activity relates to the reductions of activity we observed is not yet clear. More empirical research is needed to address these questions. It is also worth noting that most developmental and aging studies on delay discounting have focused on narrow (extreme) age ranges, and ignore individuals between 30 and 60 years of age. Future studies should close this gap and try to provide a broader perspective on the neurophysiological mechanisms underlying age-related differences in decision-making across the lifespan. At the same time, this should be complemented by theoretical approaches aimed at identifying age-related changes on a mechanistic level.

An alternative interpretation of the effects we observed could be that they reflect individual differences in choice preferences that were confounded with age, rather than age differences as such. However, an analysis of subgroups that were matched for discounting behavior did not support this account. As shown in Figure S4a, this analysis revealed significant differences in ventral striatal activity for immediate choice options in the two age groups, similar to the one observed in the whole sample (see also Figure S4b). Hence, the age-related reductions in striatal activity seem reliable even when controlling for group differences in discounting. It should be noted, however, that although age groups were matched for discounting behavior, they still differed in their overall tendency to choose for delayed reward. Hence, it could be that an additional factor other than discounting may explain the age-related differences in striatal activity. For instance, it could be that the older adults set a different reference point at which they switch to choosing delayed reward than younger adults. Another more radical alternative interpretation could be that older adults apply different cognitive mechanisms (e.g. similarity-based judgments) to solve intertemporal decision-making problems. Future studies should address these questions and control for overall differences in choice behavior while investigating discounting in different age groups.

In addition to the correlation between discounting and ventral striatal activity, we observed a significant positive correlation between discounting and reaction time on choices for delayed options. No such association was found for choices for immediate rewards (see Figure 4b). These findings suggest that individuals with a greater preference for immediate reward face increased decision difficulties on their (few) choices for delayed rewards [47]. Hence, our results are consistent with previous findings that showed higher RT and stronger activations in the delta-system for difficult decisions (choice options closer to the indifference point) as compared to easy decisions (choice options further away from the IDP) [9]. Taken together, these findings strongly support the notion that activity in the δ-system reflects the involvement of control processes during decision-making (see also Figure S1). Moreover, these results are consistent with findings that suggest that delay discounting is associated with general intelligence and that this association may be mediated by cognitive control [23].

It should be noted that the present study was not designed to differentiate between different models of delay discounting and its neural correlates, but rather as a first step to a better understanding of age-related differences in the neural systems involved in decision-making related to discounting, That is, the present findings could also be explained by assuming that the subjective value of the immediate options is lower for older than younger adults [8], and that reduced ventral striatal activity is a consequence rather than a cause of this. One explanation for such an effect could be age-related differences in income and experience with financial decisions (see [22]). One way to address this potential confound would be to adjust the choice options shown to the participants based on individual (predefined) indifference points (or discount rates) to examine age-related differences in the fMRI data for options that are matched for subjective value. Future studies using model-based fMRI analyses and more rigorous experimental designs should address this issue and try to relate age differences in discount functions more explicitly to differences in fMRI activations, while controlling for overall differences in choice behavior.

To summarize, the findings of the present study show reduced delay discounting and lower BOLD activity to immediate reward options in the ventral striatum in older than younger adults. Although both age groups show a positive association between discounting and ventral striatal activity, significant correlations were obtained in younger but not in older adults. These findings point to a reduced immediacy bias in the elderly. Furthermore, older adults show an overall increase in the percentage of delayed choices and reduced dorsal striatal activity than younger adults. This points to a reduced reward sensitivity of the dorsal striatum in older age. Hence, our findings indicate that age-related changes in decision-making are closely related and may be due to a reduced sensitivity of striatal areas to reward. These age-related changes in sensitivity to immediate reward may result from transformations in dopaminergic neuromodulation in older adults.

## Supporting Information

Figure S1 Results of the parametric RT-fMRI analysis. This analysis revealed significant correlations between RT and activity in the four major components of the delta system (dlPFC, SMA, INS and IPL) for immediate (t = 4.5, p<.0001, >20 voxels), as well as delayed choices (t = 3.6, p<.001, >20 voxels). These results show that even within individuals longer reaction times are associated with stronger activity in areas associated with cognitive control.
(EPS)

Figure S2 Contrast between immediate choice options (involving reward today) and delayed options (involving only delayed reward). A) Across age groups this contrast revealed similar, although less robust activations for immediate reward in the medial prefrontal cortex and ventro-medial prefrontal cortex. Talairach coordinates: MFG: −6, 52, 27; vmPFC: −4, 52, 2. B) The contrast also revealed significant age-related differences in the ventral striatum, similar to those obtained with the immediate vs. all choice options regressor. Talairach coordinates: vStr: −6, 2, 8.
(EPS)

Figure S3 Time courses for areas associated with the delta system (dlPFC = dorsolateral prefrontal cortex, IPL = inferior parietal lobe, Ins = Insula, SMA = supplementary motor area) and the beta system (vmPFC = ventromedial PFC, PCC = posterior cingulate cortex) shown separately for younger adults (black) and older (grey) adults. The x-axis shows time after stimulus onset in seconds, the y-axis shows percent BOLD signal change.
(EPS)

Figure S4 Subgroup analyses. A) Left: Subgroups of younger and older adults (N = 9 for each group) that were matched for discounting behavior. Right: Significant age main effect for the matched subgroups for choice options involving immediate reward in the ventral striatum (t-statistics, significant at p<.005, >20 voxels). B) Left: Subgroups of younger and older adults (N = 8 for each group) that were matched for mean percentage of delayed choices. Right: Significant age main effect for the matched subgroups for choice options involving immediate reward in the ventral striatum (t-statistics, significant at p<.001, >5 voxels).
(EPS)
